# Coming Together to Combat Tobacco's Tragic Health Consequences

**DOI:** 10.1186/1617-9625-2-5

**Published:** 2004-06-15

**Authors:** Daniel R Longo

**Affiliations:** 1 Editor-in-Chief, Columbia, Missouri, USA

## 

"Coming together is a beginning; keeping together is progress; working together is success."

- Henry Ford (1863–1947), Auto Manufacturer

Scientists and health care professionals from around the world will come together in Louisville, Kentucky, U.S., in October for the Third Annual Conference of the International Society for the Prevention of Tobacco Induced Diseases. The meeting is a further step in keeping together the effort begun by PTID founding president the late Dr. Ed Nelson in 2000. Proceedings at the meeting will help to further the Society's mission to foster scientific medical studies, health education and other efforts to prevent diseases caused by tobacco abuse, addiction, and involuntary exposure to tobacco smoke.

Louisville, located on the Ohio River in the heartland of the United States, is a historically significant city. Named for King Louis XVI of France, in recognition of his assistance during the United States' Revolutionary War, the city was founded by George Rogers Clark in 1778 – just two years after the nation declared its independence in 1776. Louisville has been home to the United States' 12th President Zachary Taylor, inventor Thomas Edison, naturalist and artist John James Audubon, and heavyweight boxing champion Muhammad Ali, among other notables.

The city boasts 11 institutions of higher learning, including the University of Louisville. With more than 21,000 students on three campuses, this university receives more than $100 million annually in grants and contracts for research. The 155-year-old University of Louisville School of Medicine is one of the oldest medical schools west of the Allegheny Mountains, and is a leader in medical research. University of Louisville surgeons, working at Jewish Hospital, successfully implanted the world's first self-contained artificial heart in 2001. In addition to the School of Medicine, the university's Health Sciences Center includes schools of Dentistry, Nursing, and Public Health and Information Sciences.

The selection of Louisville as the site for this year's PTID conference is particularly significant in light of Kentucky's prominence as a major producer of tobacco. In the U.S., Kentucky is second only to North Carolina in total production of tobacco, and leads the nation in production of burley tobacco. Tobacco accounted for 16% of Kentucky's total agricultural cash receipts in 2001. [1]

Burley tobacco, used primarily in cigarettes, is grown in 117 of Kentucky's 120 counties and accounts for 85% of the state's total tobacco production, with 220,500,000 pounds produced annually. The state also produces dark fire-cured and dark air-cured tobaccos, used in products such as snuff, chewing, and pipe tobacco. Sales of tobacco generated $566.3 million (U.S. dollars) for Kentucky farmers in 2001. [1]

But along with the economic benefits, Kentuckians have reaped the tragic health consequences that follow in tobacco's path. More than 8,000 Kentuckians die each year from illnesses caused by tobacco use. [2] More than $1.2 billion in tax dollars, through the government's Medicaid and Medicare health care programs, is spent each year to treat Kentuckians for illnesses caused or made worse by the use of tobacco products. [2]

According to the Kentucky Tobacco Prevention and Cessation Program, 32 percent of Kentucky's adults smoke; 34% of the state's high school students use cigarettes, and 15% of the state's middle school students smoke. It is estimated that 23 percent of women in Kentucky smoke during their pregnancies. [2]

In an effort to combat the negative impact of tobacco on the health of the state's residents, the Tobacco Prevention and Cessation Program has adopted four goals established by the U.S. Centers for Disease Control: preventing the initiation of tobacco use among young people; promoting cessation among young people and adults; eliminating non-smokers' exposure to environmental tobacco smoke; and identifying and eliminating disparities related to tobacco and its effects on different population groups. [2]

The presence of PTID scientists and health care professionals in Louisville will be an encouragement to Kentuckians who are working to address the problem of tobacco-induced diseases in their state.

Dates for the PTID annual conference are October 29–November 1, 2004. The conference will be held at the Seelbach Hilton in Louisville. More information is available on the conference website, .

If you have not yet made plans to attend this conference, we encourage you to do so. Coming together, keeping together, and working together, we can make a difference for millions of adults and young people who are at risk for tobacco-induced disease and death.

## Competing interests

The authors declare that they have no competing interests.
